# Long-term mepolizumab treatment reduces relapse rates in super-responders with eosinophilic granulomatosis with polyangiitis

**DOI:** 10.1186/s13223-023-00801-7

**Published:** 2023-05-13

**Authors:** Nami Masumoto, Chiyako Oshikata, Ryo Nakadegawa, Yuto Motobayashi, Reeko Osada, Saki Manabe, Takeshi Kaneko, Naomi Tsurikisawa

**Affiliations:** 1 Department of Respirology, National Hospital Organization Yokohama Medical Center, 3-60-2 Harajuku, Totsuka-Ku, Yokohama, 245-8575 Japan; 2 Department of Pulmonology, Yokohama City University Graduate School of Medicine, 3-9 Fukuura, Kanazawa-Ku, Yokohama, Kanagawa 236-0004 Japan; 3 Department of Allergy and Respirology, Hiratsuka City Hospital, 1-19-1 Minamihara, Hiratsuka, Kanagawa 254-0065 Japan

**Keywords:** Churg–Strauss syndrome, Eosinophilic granulomatosis with polyangiitis, Intravenous immunoglobulin, Mepolizumab, Systemic vasculitis

## Abstract

**Background:**

The mainstay of treatment for eosinophilic granulomatosis with polyangiitis (EGPA) is systemic corticosteroid therapy; some patients also receive intravenous immunoglobulins, other immunosuppressive agents, and biologics. Mepolizumab, an anti-interleukin-5 monoclonal antibody, induces remission and decreases the daily corticosteroid dose; however, the clinical efficacy of mepolizumab in EGPA and the prognosis with long-term treatment with this drug are unknown.

**Methods:**

Seventy-one EGPA patients were treated at Hiratsuka City Hospital, Japan, between April 2018 and March 2022. We administered mepolizumab for a mean of 2.8 ± 1.7 years to 43 patients in whom remission could not be induced by conventional treatment. After excluding 18 patients who had received mepolizumab for less than 3 years, we classified 15 patients into a “super-responder group” (the daily dose of corticosteroids or other immunosuppressant could be decreased, or the interval between IVIG treatments could be prolonged) and 10 patients into a “responder group” (neither of these changes was achievable). Eosinophil numbers, serum IgG levels, daily doses of corticosteroids and other immunosuppressants, Birmingham Vasculitis Activity Score (BVAS), and relapse frequency before and after mepolizumab initiation were determined.

**Results:**

Blood eosinophil count at diagnosis and the lowest serum IgG level before mepolizumab treatment were significantly higher in super-responders than in responders (*p* < 0.05). In super-responders, the prednisolone dose at last visit on mepolizumab treatment was lower than that before treatment (*p* < 0.01) and lower than that at last visit in the responders (*p* < 0.01). In both groups, peripheral blood eosinophil numbers and BVAS were lower after starting mepolizumab than before (*p* < 0.01). BVAS before mepolizumab (*p* < 0.05) and at last visit (*p* < 0.01) were lower in super-responders than in responders. Relapse rates every year after the start of mepolizumab were lower in super-responders than in responder groups (*p* < 0.01). In super-responders, relapse rates were lower during the 3 years following mepolizumab initiation (*p* < 0.01) and at last visit (*p* < 0.01) were significantly lower than after 1 year of treatment.

**Conclusion:**

Mepolizumab treatment of super-responders sustainably reduced the relapse rate.

## Background

Eosinophilic granulomatosis with polyangiitis (EGPA) is a rare immunological condition characterized by allergic granulomatosis and small- and medium-vessel necrotizing vasculitis associated with peripheral blood eosinophilia and tissue infiltration by eosinophils [1, 2]. Eosinophils are the most important effector cells and contribute to the disease mechanism [2, 3]. Interleukin (IL)-5, which regulates eosinophil proliferation, maturation, and differentiation, is produced by T-helper (Th)2 cells and type 2 innate lymphoid cells (ILC2s) [4, 5]. Serum IL-5 or IL-25 levels are more highly correlated with disease activity in patients with active EGPA than in those with asthma [6, 7], and in these patients they are significantly higher than in healthy controls [8]. We reported previously that the serum IL-33 concentration and peripheral blood ILC2 count increase in patients with active EGPA at diagnosis and relapse [9].

The mainstay of treatment for EGPA is systemic corticosteroid therapy; some patients receive additional treatment with other immunosuppressive agents, such as cyclophosphamide, azathioprine, methotrexate, interferon-α [10, 11], rituximab [12–14], intravenous immunoglobulins (IVIGs) [15–17], and other biologics [18, 19]. Biologics include omalizumab [20, 21], mepolizumab [21–28], benralizumab [29] dupilumab [30], reslizumab, and lebrikizumab; the latter three are being tested in ongoing clinical trials [31].

Mepolizumab, an anti-IL-5 monoclonal antibody, reduces blood eosinophil counts, and its efficacy in severe asthma has been established in large-scale trials [26, 27]. In 2010, mepolizumab administered by 750-mg intravenous infusion every 4 weeks was shown to reduce the peripheral eosinophil count effectively and to be safe and well tolerated in patients with EGPA [22]. In addition to decreasing the peripheral eosinophil count, mepolizumab improves the signs of vasculitis, such as asthma exacerbation, sinusitis, arthralgia, multiple polyneuropathy, gastrointestinal tract involvement, and skin involvement [28, 32], and its use reduces the daily systemic corticosteroid dose in patients with refractory or relapsed EGPA [23, 24]. In 2017, Mepolizumab In Relapsing or Refractory EGPA (MIRRA)—the first randomized, double-blind, placebo-controlled trial of a subcutaneous 300-mg monthly dose of mepolizumab for patients with relapsing or refractory EGPA—led to a significant increase in remission duration in the proportion of patients achieving remission. Moreover, remission occurred in 53% of participants in the mepolizumab group vs. 19% in the placebo group, and at the end of the study the average daily prednisolone (PSL) dose had been reduced to only 4 mg in 44% of participants in the mepolizumab treatment group [25]. This was the first demonstration of the efficacy of mepolizumab in maintaining remission in EGPA.

Details of the specific effects of mepolizumab on vasculitis symptoms were not fully analyzed in the MIRRA study [25]. However, in a later study, we found that, among EGPA patients treated with mepolizumab, there are super-responders displaying marked effects and responders displaying weak effects. We found that mepolizumab reduced vasculitis symptoms in many organs, and that the efficient response to mepolizumab in super-responders was correlated with high peripheral blood eosinophil counts at diagnosis and high serum IgG levels before mepolizumab administration [28].

Since 1994, prognosis and mortality rates for EGPA have improved as a result of better diagnosis in accordance with American College of Rheumatology criteria [33] and improvements in treatment over the last 20 years. EGPA patients diagnosed after 1996 have a better prognosis than those diagnosed before 1996 [34]. However, patients with Five Factor Score 2009 criteria of age ≥ 65 years, severe cardiac involvement, severe gastrointestinal tract involvement, severe renal insufficiency, and status without sinusitis ≥ 2 still have a poor prognosis [35]. Additionally, the 5−, 10−, and 20-year survival rates reported in 2011 by Guillevin et al. were about 90%, 75%, and 45%, respectively [35], whereas those reported in 2013 by Moosig et al. were 97%, 89%, and 72%, respectively [36]; we reported in 2017 that these survival rates were 91.1%, 83.7%, and 68.6%, respectively [15]. EGPA is thus a disease with a poor prognosis in the long term despite the recent improvements.

Long-term biologic treatment of bronchial asthma has been reported only for omalizumab, which has now been administered for as long as 10 years [37]. Other biologics used in asthma, such as mepolizumab [38], reslizumab [38, 39], and benralizumab [38, 40], have been administered for about 2 years. In contrast, mepolizumab is the only biologic available in Japan for EGPA, and until now there have been no reports of long-term analysis or follow-up.

The clinical efficacy of mepolizumab in EGPA and the disease prognosis with long-term treatment with this drug are unknown. Here, we selected EGPA patients who had received mepolizumab for at least 3 years, and we analyzed the differences in background factors between super-responders and responders. We report the frequency of relapse per year in patients in each group and discuss whether treatment of EGPA with mepolizumab affects the prognosis of the disease.

## Methods

### Patients

We retrospectively collected data on the clinical courses of all 71 patients with EGPA treated at the Department of Allergy and Respirology, Hiratsuka City Hospital, Kanagawa, Japan, from April 2018 through March 2022. EGPA was diagnosed according to allergic granulomatosis angiitis (known as Churg–Strauss syndrome) criteria [41] and classified by using the American College of Rheumatology criteria [33]. Diagnosis was contingent on the presence of at least four of the following six features: eosinophilia, asthma, pulmonary infiltrate, polyneuropathy, extravascular eosinophils, and paranasal sinus abnormality. Human Subject Protection Committee approval at Hiratsuka City Hospital (30-013) was obtained for this retrospective review of existing medical records.

### Study design

**Fig. 1:**
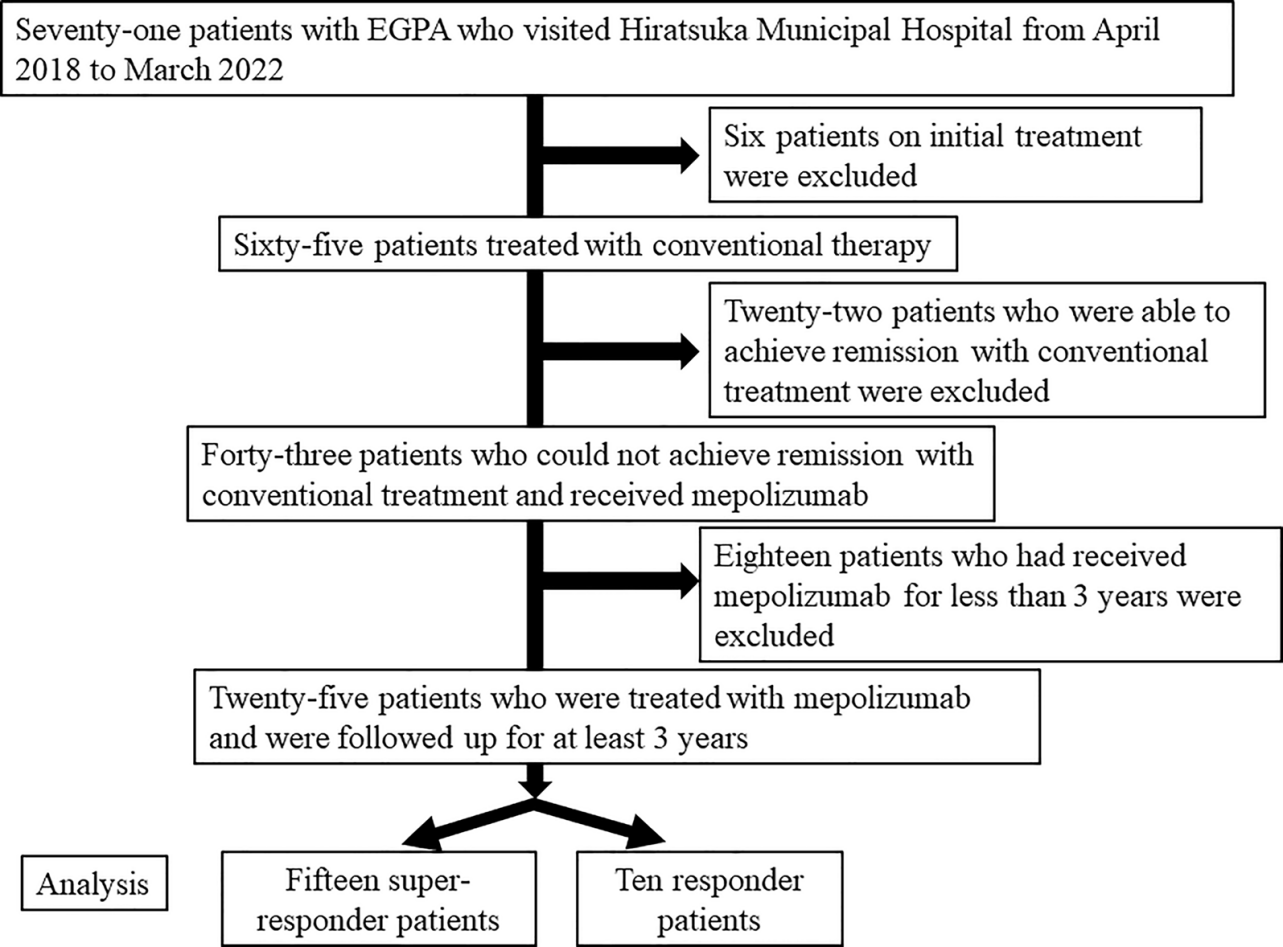
Protocol and follow-up for patients with EGPA who were treated with mepolizumab for at least 3 years. The initial group consisted of 71 patients with EGPA who had visited Hiratsuka City Hospital from April 2018 to March 2022. We excluded six patients on initial treatment and 22 who had achieved remission with conventional treatment. We then analyzed the data on the remaining 25 patients treated with mepolizumab who were followed up for at least 3 years. We analyzed the clinical characteristics of responders and super-responders separately

Of the 71 EGPA patients, we excluded six who were undergoing initial treatment and 22 who had achieved remission with conventional treatment (e.g., corticosteroids, cyclophosphamide, azathioprine, and methotrexate). We administered mepolizumab to the remaining 43 patients, in whom remission could not be induced by conventional treatment. However, we then excluded 18 patients who had received mepolizumab for less than 3 years. The remaining 25 patients were classified into two groups: 15 in a “super-responder group” (the daily dose of corticosteroid or another immunosuppressant was able to be decreased, or the interval between IVIG treatments was able to be prolonged) and 10 in a “responder group” (for whom neither of these changes could be achieved; Fig. 1).

We defined the relapse rate as the total number of relapses per year occurring before and after mepolizumab treatment-group allocation. A state of remission was defined as the absence of clinical signs or symptoms of active vasculitis after initial treatment. A state of relapse was defined as the recurrence, after remission, of vasculitis symptoms and signs, excluding the exacerbation of asthma or sinusitis (with or without an increase in the proportion of eosinophils among white blood cells) [42], that required the resumption of immunosuppressive therapy or an increased dose of immunosuppressant. A large increase in tissue eosinophilia was potentially associated with increased severity of vasculitis signs and symptoms, but this criterion was not considered in our definition of relapse. We used the manual muscle test scored on the Medical Research Council scale (0–5) and electromyographic examination to evaluate motor nerve dysfunction presenting as mononeuritis multiplex. We assessed sensory nerve dysfunction subjectively from symptoms and physical examination. Lung involvement was defined as consolidation with ground-glass-opacity-containing nodules, interlobular septal thickening [43, 44], lymph node enlargement, thickening of the bronchial wall [45, 46], pleural effusion (identified by high-resolution computed tomography [43, 47]), or infiltration by eosinophils (detected by lung biopsy). We defined cardiac involvement as cardiac symptoms (chest discomfort or pain, palpitations, or back pain) or abnormal signs (assessed by Holter electrocardiogram, cardiac echocardiography, plasma B-type natriuretic peptide quantification, or ^123^I-metaiodobenzylguanidine myocardial imaging) in all patients. We defined gastrointestinal involvement as symptoms of epigastric pain, abdominal pain, constipation, or diarrhea, or positive endoscopic signs, with biopsy detection of gastrointestinal eosinophil infiltration or edematous colonic submucosal changes [48]. Erythema, purpura, livedo, acrocyanosis, ulceration, nodule formation, or biopsy detection of eosinophil infiltration was considered to indicate skin involvement. Headache, abnormal visual sensation or visual disorder, cerebral infarction or bleeding, and cranial nerve dysfunction were taken as indicators of central nervous system involvement. We defined renal involvement as the presence of any of the following: urinary eosinophils, glomerulonephritis, nephrosis (proteinuria > 3.5 g/day), proteinuria (> 0.5 g/day or > 50 mg/dL), or renal dysfunction (creatinine > 20% of baseline). An otorhinolaryngologist diagnosed otitis media in all patients with ear symptoms such as otorrhea, ear fullness, and hearing loss.

Organs compromised by asthma or sinusitis were not included among the total number of organs involved. We used the Birmingham Vasculitis Activity Score (BVAS) [49] to assess disease activity from the time of diagnosis to before mepolizumab treatment initiation and at the time of the last visit while on mepolizumab. The BVAS evaluates nine categories of symptoms and signs: systemic; cutaneous; ear, nose, and throat; mucous membranes and eyes; heart and vessels; chest; renal system; gastrointestinal tract; and nervous system. The maximum number of possible points scorable in each category is 7, making the total maximum score 63.

We assayed peripheral blood white blood cell and eosinophil counts and serum myeloperoxidase–anti-neutrophil cytoplasmic antibody (MPO-ANCA), proteinase 3 (PR3)-ANCA, immune complex, and IgG levels at disease diagnosis in all EGPA patients. We determined the lowest serum IgG levels from pretreatment to the last visit. Initial treatment of all patients with pulsed steroids, initial doses of PSL, and initial treatment with immunosuppressants (cyclophosphamide, azathioprine, methotrexate, cyclosporine, and rituximab) were determined from the medical records. Before and after group allocation, maintenance treatment with the above immunosuppressants, the maintenance dose of PSL, and treatment with IVIG (Venilon, Teijin, Tokyo, Japan; 400 mg/kg daily for 5 days) were also determined from the medical records. The study endpoint was the last examination during mepolizumab administration performed within the study period. We evaluated eosinophil counts in the peripheral blood at diagnosis, before and every year after mepolizumab initiation, and at the last examination before the end of the study. We determined the BVAS at the time of diagnosis, before mepolizumab treatment-group allocation, every year during administration of mepolizumab, and at the final visit within the study period. In the super-responder and responder groups, we investigated relapse rates from the time of diagnosis to 1 year or more before mepolizumab administration, within the year before the start of mepolizumab administration, and every year after initiation of mepolizumab administration until the last medical examination during mepolizumab treatment. We analyzed the daily dose of PSL initially, before treatment-group allocation, every year during mepolizumab treatment, and at the last examination during mepolizumab treatment.

## Results

### Clinical manifestations of patients treated or not treated with mepolizumab

Age at onset of asthma or EGPA and the period from EGPA diagnosis until study admission did not differ between patients treated or not treated with mepolizumab (Table 1). The numbers of white blood cells and peripheral blood eosinophils and the percentage positivities for MPO-ANCA and PR3-ANCA, and serum levels of IgE and IgG at diagnosis did not differ between patients treated and not treated with mepolizumab. Most clinical manifestations at diagnosis and BVAS at diagnosis did not differ between the two groups; the exception was myocardial involvement at diagnosis, which was significantly more prevalent in patients who received mepolizumab than in those who did not (*p* < 0.01). BVAS and the relapse rate before mepolizumab-treatment-group allocation were significantly higher in patients who received mepolizumab than in those who did not (*p* < 0.01). The use of pulsed steroids, the daily dose of PSL, and the use of immunosuppressant therapy at initial treatment did not differ between patients treated or not treated with mepolizumab. Use of IVIG (*p* < 0.05) and the maintenance dose of PSL (*p* < 0.05) before group allocation were significantly higher in the mepolizumab group than in those not allocated to receive mepolizumab (Table 1).

**Table 1:** Characteristics of 71 patients with EGPA

	Mepolizumab group	Conventional treatment group	*p-*value
	N = 43	N = 28	
Age at study admission (years), mean ± 1 SD	59.9 ± 14.1	56.4 ± 16.3	NS
Sex (M/F)	16/27	9/19	NS
Age at asthma onset (years), mean ± SD	41.3 ± 20.0	34.4 ± 17.9	NS
Age at EGPA onset (years), mean ± SD	50.4 ± 18.4	49.4 ± 15.2	NS
Period of time from EGPA onset to study admission (years), mean ± SD	8.4 ± 8.7	6.0 ± 5.4	NS
Laboratory data at diagnosis			
WBC count (/μL), mean ± 1 SD	16 220.9 ± 8103.7	15 508.2 ± 9173.2	NS
Blood eosinophils (/μL), mean ± 1 SD	7877.2 ± 7018.9	7515.8 ± 7461.6	NS
MPO-ANCA yes/no (%)	10/33 (23.3%)	6/22 (21.4%)	NS
PR3-ANCA yes/no (%)	3/40 (7.0%)	4/24 (14.3%)	NS
IC > 1.5 mg/mL yes/no (%)	6/26 (25.7%)	6/15 (28.6%)	NS
RF > 15 IU/mL yes/no (%)	14/22 (38.9%)	14/11 (56.0%)	NS
BNP > 18.7 pg/mL yes/no (%)	19/18 (51.4%)	11/10 (52.4%)	NS
IgE IU/mL, median (range)	432 (56–3176)	542 (11.4–25,112)	NS
IgG IU/mL, mean ± SD yes/no (%)	1562.4 ± 609.5	1441.7 ± 503.6	NS
Clinical manifestations at diagnosis, yes/no (%)			
Asthma	43/0 (100.0%)	27/1 (96.4%)	NS
Paranasal sinusitis	41/2 (95.3%)	25/3 (89.3%)	NS
Eosinophilic otitis media	12/30 (28.6%)	4/23 (14.8%)	NS
Multiple polyneuropathy	42/1 (97.7%)	28/0 (100%)	NS
Minimum MMT score	3.6 ± 0.9	3.3 ± 1.1	NS
Pulmonary involvement	21/18 (53.8%)	14/14 (50.0%)	NS
Myocardial involvement	33/9 (78.6%)	12/14 (46.2%)	p < 0.01
Gastrointestinal tract involvement	37/2 (94.9%)	23/3 (88.5%)	NS
Liver, gallbladder, pancreas	8/31 (20.5%)	8/18 (30.8%)	NS
Renal involvement	14/27 (34.1%)	7/21 (25.0%)	NS
Skin involvement	27/14 (65.9%)	15/13 (53.6%)	NS
Arthralgia	23/19 (54.8%)	11/17 (39.3%)	NS
Myalgia	8/33 (19.5%)	3/24 (11.1%)	NS
Central nervous system involvement	12/31 (27.9%)	9/19 (32.1%)	NS
BVAS			
At diagnosis	31.0 ± 7.3	28.3 ± 11.0	NS
Before treatment-group allocation	15.4 ± 5.2	7.3 ± 6.4	< 0.01
Relapses before treatment-group allocation			
Frequency (times/year), mean ± 1 SD	1.0 ± 0.9	0.4 ± 0.6	< 0.01
Initial treatment			
PSL (mg) (mean ± SD)	42.9 ± 11.7	46.4 ± 11.1	NS
mPSL pulse yes/no (%)	29/14 (67.4%)	22/6 (78.6%)	NS
Immunosuppressant yes/no (%)	37/6 (86.0%)	22/6 (78.6%)	NS
CYC/AZA/CsA/MTX/RTX	25/3/6/3/0	10/3/7/2/0	NS
Maintenance treatment before group allocation			
Immunosuppressant yes/no (%)	33/10 (76.7%)	21/7 (75.0%)	NS
CYC/AZA/CSA/MTX/RTX	7/6/5/13/2	6/4/7/4/0	NS
PSL (mg) (mean ± SD)	12.3 ± 5.6	8.8 ± 5.0	< 0.05
IVIG yes/no (%)	35/8 (81.4%)	16/12 (57.1%)	< 0.05

*AZA* Azathioprine, *BNP* Brain natriuretic peptide, *BVAS* Birmingham vasculitis activity score, *CsA* Cyclosporine A, *CYC* Cyclophosphamide, *EGPA* Eosinophilic granulomatosis with polyangiitis, *IC* Immune complex, *IVIG* Intravenous immunoglobulin, *MMT* Manual muscle test, *MPO-ANCA* Myeloperoxidase antineutrophil cytoplasmic antibodies, *mPSL* meTHylprednisolone, *MTX* Methotrexate, *PR3* Protein 3, *PSL* Prednisolone, *RF* Rheumatoid factor, *RTX* Rituximab

### Time to onset of effect after mepolizumab initiation

**Fig. 2:**
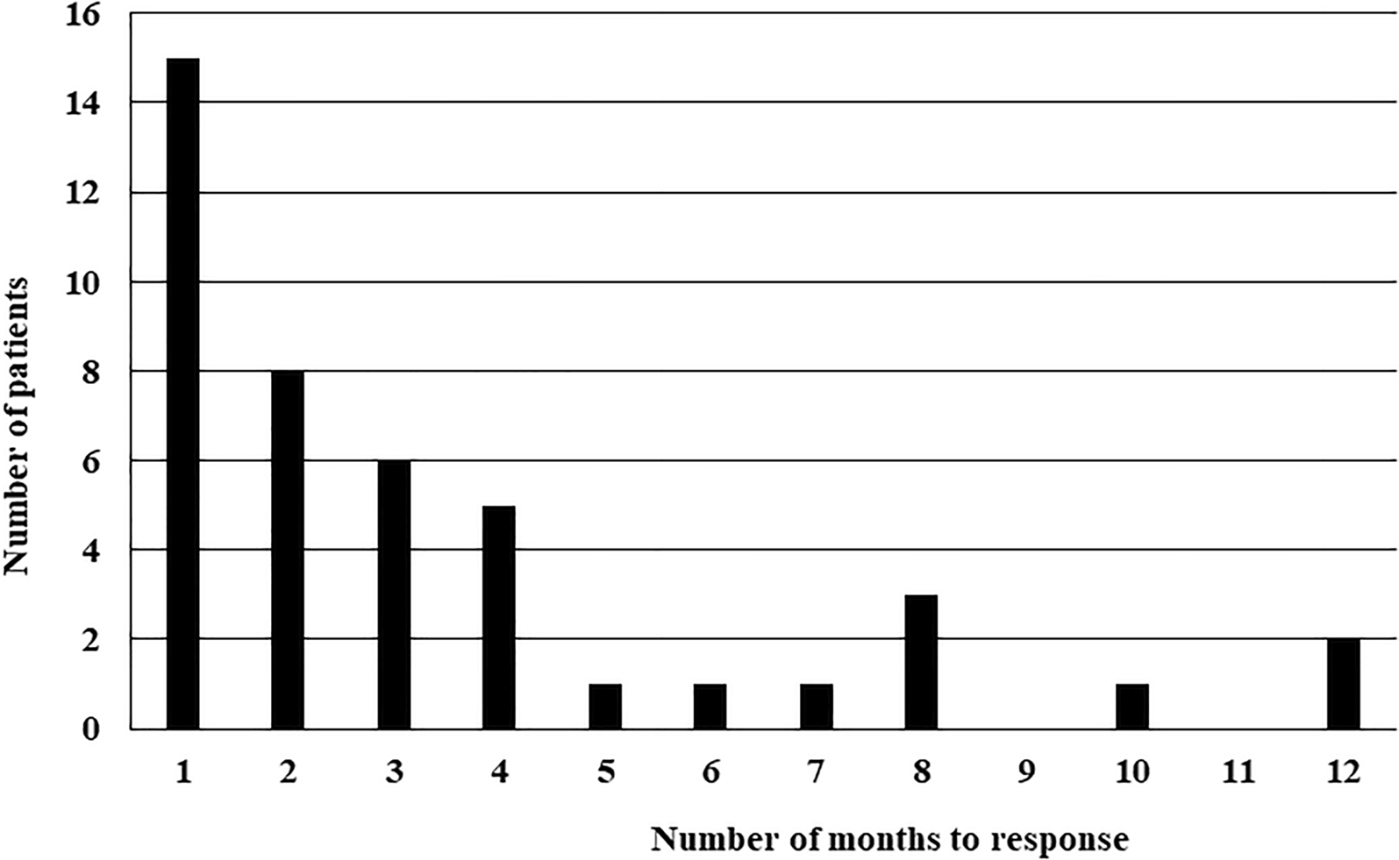
Analysis of time after administration of mepolizumab to onset of effect. After administering mepolizumab, we confirmed some clinical benefit in all 43 patients. We found clinical efficacy of mepolizumab 3.4 ± 3.0 months after administration (mean ± SD); for 15 patients (34.9%), efficacy was observed within 1 month

We administered mepolizumab for a mean of 2.8 ± 1.7 years to 43 patients in whom remission could not be induced by conventional treatment. Vasculitis-related signs improved in all 43 patients who received mepolizumab. The period of time from the start of administration of mepolizumab to the improvement of clinical signs and symptoms varied widely (Fig. 2). After the start of mepolizumab treatment, a clinical response was observed after an average of 3.4 ± 3.0 months, and 15 out of 43 patients (34.9%) had a clinical response within 1 × month. There were also cases in which improvement of clinical symptoms was observed only after 6 months to 1 year.

### Comparison of patient characteristics between super-responders and responders

**Fig. 3:**
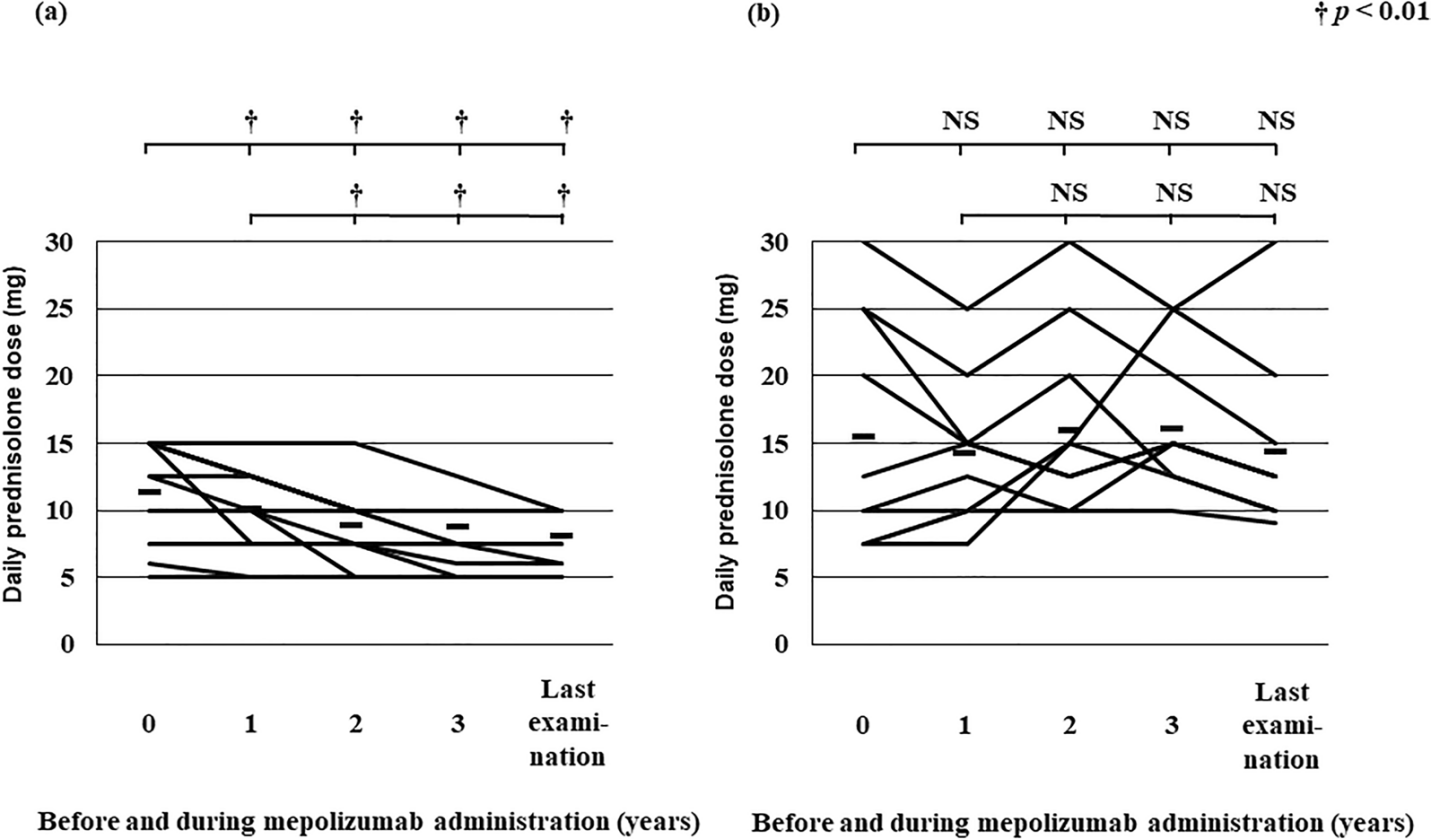
Daily dose of prednisolone from before mepolizumab initiation to the time of the last visit while still on mepolizumab (mean of 3.4 ± 3.0 months after initiation) in EGPA patients in the super-responder group (**a**) and the responder group (**b**). Dashes lines show mean dose of prednisolone. Mean values for each patient group were compared by using the Wilcoxon matched-pairs *t*-test; *p* < 0.05 was considered statistically significant. †*p* < 0.01; NS, not significant. Upper brackets at the top show the significance of differences between initiation of mepolizumab treatment versus years 1, 2, 3, and last examination during treatment; lower brackets at the top show the significance of differences between 1 year after initiation of mepolizumab treatment versus years 2, 3, and the last examination during treatment

The initial dose of PSL and the maintenance dose before mepolizumab initiation did not differ between the super-responder and responder groups, but the PSL dose at the time of the last examination on mepolizumab was significantly lower in the super-responders than in the responders (*p* < 0.01; Table 2). In the super-responder group, the PSL dose was significantly lower at all time points after mepolizumab initiation than before treatment (*p* < 0.01); moreover, the dose at the time of the last examination was still significantly lower than the initial dose (Fig. 3a). The PSL dose after mepolizumab treatment initiation also declined, but not significantly so, in a few of the patients in the responder group (Fig. 3b). The percentage use of immunosuppressant was significantly lower in the super-responder group than in the responder group before mepolizumab initiation (*p* < 0.05), but at the last visit there was no significant difference in this parameter between the two groups (Table 2).

**Table 2:** Characteristics of 25 EGPA patients treated with mepolizumab for at least 3 years

	Super-responders	Responders	*p-*value
	N = 15	N = 10	
Age at study admission (years), mean ± SD	59.1 ± 10.9	58.9 ± 11.7	NS
Sex (M/F)	8/7	2/8	NS
Age at asthma onset (years), mean ± SD	34.1 ± 16.9	39.4 ± 19.6	NS
Age at EGPA onset (years), mean ± SD	42.6 ± 16.1	45.9 ± 13.3	NS
Period of time from EGPA onset to study admission (years), mean ± SD	16.3 ± 8.7	13.2 ± 8.8	NS
Laboratory data at diagnosis			
WBC count (/μL), mean ± SD	18 195.3 ± 7045.1	13 232.9 ± 9150.7	< 0.05
Blood eosinophils (/μL), mean ± SD	10 580.4 ± 6370.4	6935.3 ± 7549.5	< 0.05
MPO-ANCA yes/no (%)	6/9 (40.0)	2/8 (20.0)	NS
PR3-ANCA yes/no (%)	1/14 (6.7)	0/10 (0)	NS
IC > 1.5 mg/mL yes/no (%)	3/8 (27.3)	2/5 (28.6)	NS
RF > 15 IU/mL yes/no (%)	5/7 (41.7)	3/4 (42.9)	NS
BNP > 18.7 pg/mL yes/no (%)	3/9 (25.0)	3/4 (42.9)	NS
IgE (IU/mL), median (range)	545 (35–4950)	342 (34–28,403)	NS
IgG (IU/mL), mean ± SD	1612.4 ± 747.9	1802.1 ± 684.0	NS
Blood eosinophil count			
Before mepolizumab (/μL), mean ± SD	203.2 ± 267.9	78.6 ± 125.8	NS
At last examination (/μL), mean ± SD	14.2 ± 36.9	7.6 ± 14.9	NS
Lowest serum IgG before administration of mepolizumab (IU/mL), mean ± SD	801.7 ± 194.0	608.7 ± 211.0	< 0.05
Clinical manifestations at diagnosis, yes/no (%)			
Asthma	15/0 (100)	10/0 (100.0)	NS
Paranasal sinusitis	15/0 (100)	9/1 (90.0)	NS
Eosinophilic otitis media	4/11 (26.7)	4/6 (40.0)	NS
Multiple polyneuropathy	14/1 (93.3)	10/0 (100)	NS
Minimum MMT score	3.8 ± 0.8	3.3 ± 0.9	NS
Pulmonary involvement	6/7 (46.2)	4/5 (44.4)	NS
Myocardial involvement	11/4 (73.3)	9/1 (90.0)	NS
Gastrointestinal tract involvement	13/2 (86.7)	8/2 (80.0)	NS
Liver, gall bladder, pancreas	2/11 (15.4)	3/9 (33.3)	NS
Renal involvement	6/9 (40.0)	4/6 (40.0)	NS
Skin involvement	10/4 (71.4)	8/1 (88.9)	NS
Arthralgia	7/8 (46.7)	8/1 (88.9)	< 0.05
Myalgia	2/12 (14.3)	5/4 (55.6)	< 0.05
Central nervous system involvement	5/10 (33.3)	3/7 (30.0)	NS
BVAS			
At diagnosis, mean ± SD	32.3 ± 5.9	35.5 ± 7.6	NS
Before mepolizumab, mean ± SD	15.1 ± 4.9	20.2 ± 4.6	< 0.05
At last visit on mepolizumab, mean ± SD	5.2 ± 5.3	13.0 ± 5.0	< 0.01
Relapse rates before mepolizumab treatment (times/year), mean ± SD			
Frequency of relapse from diagnosis to 1 year or more before mepolizumab	0.4 ± 0.4	1.0 ± 0.8	NS
Frequency of relapse within 1 year before mepolizumab initiation	2.2 ± 0.8	2.8 ± 0.9	NS
Frequency of relapse 1 year after mepolizumab initiation	1.1 ± 0.6	3.7 ± 2.1	< 0.01
Frequency of relapse 2 years after mepolizumab initiation	0.8 ± 0.7	3.1 ± 2.3	< 0.01
Frequency of relapse 3 years after mepolizumab initiation	0.6 ± 0.6	3.5 ± 2.4	< 0.01
Frequency of relapse from mepolizumab initiation to final visit	0.4 ± 0.4	3.4 ± 2.4	< 0.01
Initial treatment			
Pulsed mPSL yes/no (%)	10/5 (66.7)	5/5 (50.0)	NS
PSL (mg), mean ± SD	48.0 ± 12.1	41.5 ± 8.8	NS
Immunosuppressant yes/no (%)	13/2 (86.7)	10/0 (100)	NS
CYC/AZA/CsA/MTX/RTX	9/1/2/1/0	6/1/2/1/0	NS
Maintenance treatment before mepolizumab			
Immunosuppressant yes/no (%)	10/5 (66.7)	10/0 (100.0)	< 0.05
CYC/AZA/CsA/MTX/RTX	0/2/2/6/0	0/2/0/6/2	NS
PSL (mg), mean ± SD	11.7 ± 3.7	15.5 ± 8.6	NS
IVIG yes/no (%)	12/3 (80.0)	10/0 (100)	NS
Duration of mepolizumab administration (years), mean ± SD	4.3 ± 1.0	4.2 ± 1.0	NS
Maintenance treatment at last visit on mepolizumab			
Immunosuppressant yes/no (%)	11/4 (73.3)	10/0 (100.0)	NS
CYC/AZA/CsA/MTX/RTX	0/2/2/7/0	0/3/0/4/3	NS
PSL (mg), mean ± SD	7.6 ± 2.2	14.2 ± 6.4	< 0.01

*AZA* Azathioprine, *BNP* Brain natriuretic peptide, *BVAS* Birmingham vasculitis activity score, *CsA* Cyclosporine A, *CYC* Cyclophosphamide, *EGPA* Eosinophilic granulomatosis with polyangiitis, *IC* Immune complex, *IVIG* Intravenous immunoglobulin, *MMT* Manual muscle test, *MPO-ANCA* Myeloperoxidase antineutrophil cytoplasmic antibodies, *mPSL* Methylprednisolone, *MTX* mEthotrexate, *PR3* Protein 3, *PSL* Prednisolone, *RF* Rhumatoid factor, *RTX* Rituximab

**Fig. 4:**
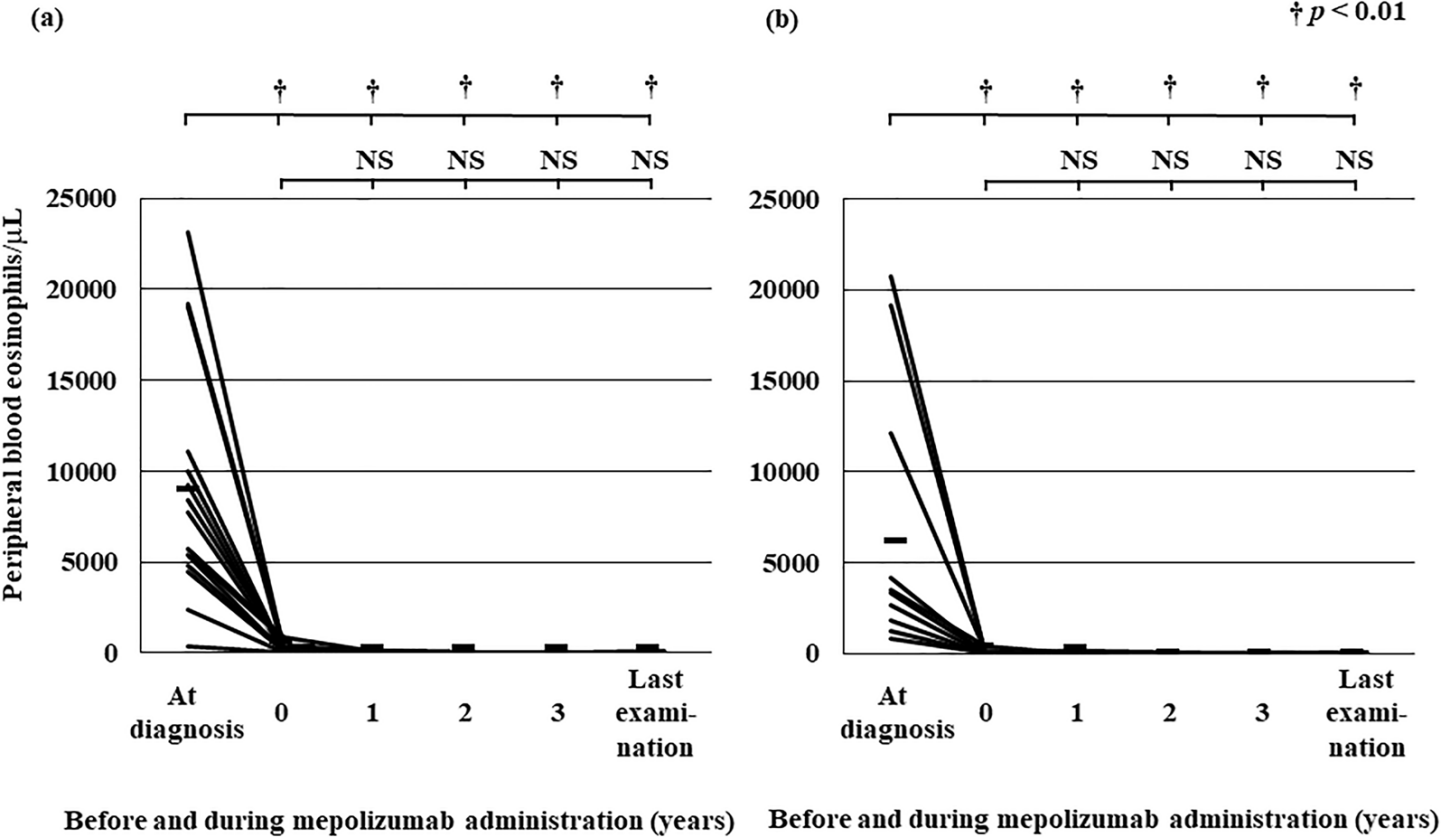
Peripheral blood eosinophil counts throughout the course of treatment, from the time of disease diagnosis in the super-responder group of EGPA patients (**a**) and the responder group (**b**). Each line represents the mean eosinophil count. Mean values for each patient group were compared by using the Wilcoxon matched-pairs *t*-test; *p* < 0.05 was considered statistically significant. †*p* < 0.01; NS, not significant. Upper brackets at the top show the significance of differences between time of diagnosis versus time of mepolizumab treatment initiation and years 1, 2, 3, and last examination during mepolizumab treatment; lower brackets show the significance of differences between time of initiation of mepolizumab treatment versus years 1, 2, 3, and last examination

Age at onset of asthma or EGPA and period of time from EGPA diagnosis until study admission did not differ significantly between the super-responder and responder groups (Table 2). The numbers of peripheral blood white blood cells and eosinophils at diagnosis were significantly higher in the super-responder group than in the responder group (*p* < 0.05), but the number of eosinophils did not differ between groups before the administration of mepolizumab, after the start of mepolizumab treatment, or at the last examination (Table 2). In both super-responders and responders, the number of peripheral blood eosinophils decreased significantly between diagnosis and initiation of mepolizumab treatment (*p* < 0.01), as well as between diagnosis and every year after initiation of mepolizumab treatment and the time of the last examination during mepolizumab treatment (*p* < 0.01). However, within each group, there were no differences in eosinophil count between before mepolizumab treatment and the time of the last examination (Fig. 4a, b).

**Fig. 5:**
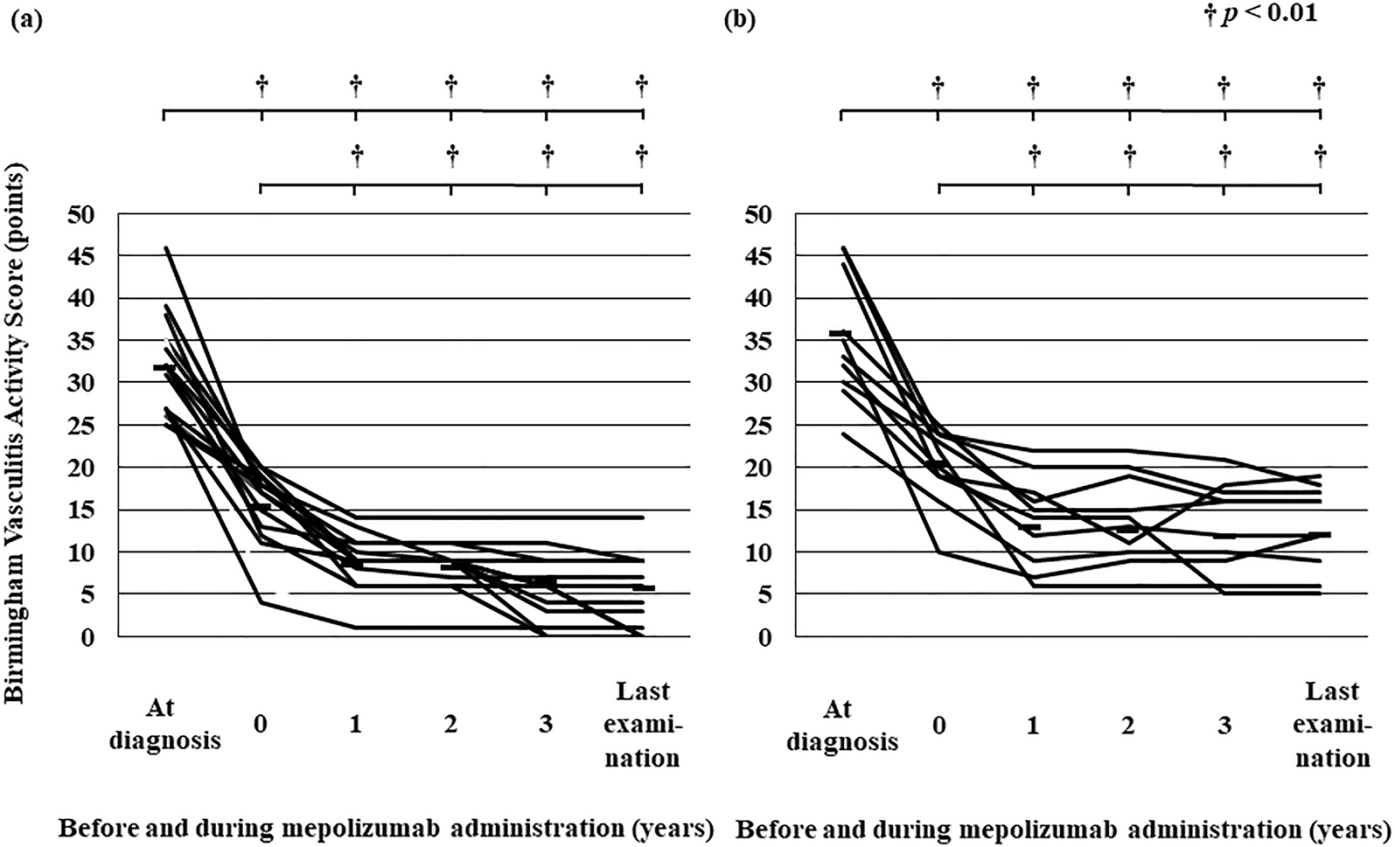
Birmingham Vasculitis Activity Scores from the time of disease diagnosis to before mepolizumab treatment initiation, during treatment, or at the last visit while on mepolizumab, in EGPA patients in the super-responder group (**a**) and the responder group (**b**). Each line represents mean scores. Mean values for each patient group were compared by using the Wilcoxon matched-pairs *t*-test; *p* < 0.05 was considered statistically significant. †*p* < 0.01. Upper brackets at the top show the significance of differences between diagnosis versus initiation of mepolizumab treatment and years 1, 2, 3, and last examination during mepolizumab treatment; lower brackets show the significance of differences between initiation of mepolizumab treatment versus years 1, 2, 3, and last examination

**Fig. 6:**
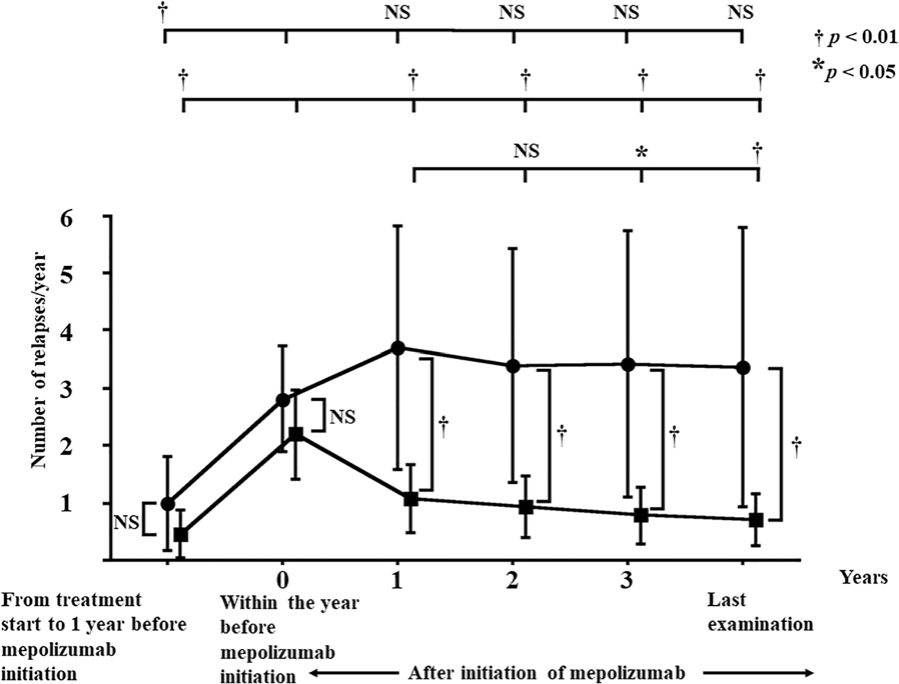
Frequency of relapse (times/year) from the start of conventional treatment to 1 year before mepolizumab administration, within the year before mepolizumab initiation, and by year until the final visit in the super-responder group (squares) and the responder group (circles). Mean values for responder and super-responder groups were compared by using the Wilcoxon matched-pairs *t*-test; *p* < 0.05 (*) was considered statistically significant. †*p* < 0.01. Upper brackets at the top show the significance of differences between the start of conventional treatment versus 1 year before initiation of mepolizumab treatment, the time of initiation of mepolizumab treatment, and years 1, 2, 3, and last examination during mepolizumab treatment in the responder group; middle brackets show the significance of differences between the start of conventional treatment versus 1 year before initiation of mepolizumab treatment, the time of initiation of mepolizumab treatment, and years 1, 2, 3, and last examination during mepolizumab treatment in the super-responder group; lower brackets show the significance of differences between 1 year after initiation of mepolizumab and years 2 and 3 and last examination during mepolizumab treatment in the super-responder group

At diagnosis, almost all clinical manifestations did not differ between the super-responder and responder groups. However, rates of arthralgia and myalgia were higher in the responder group than in the super-responder group (*p* < 0.05; Table 2). BVAS at diagnosis did not differ between super-responders and responders, but BVAS both before treatment with mepolizumab (*p* < 0.05) and at last examination (*p* < 0.01) was significantly higher in responders than in super-responders (Table 2). In both groups, BVAS decreased significantly from the time of diagnosis to before treatment with mepolizumab (*p* < 0.01), as well as from before mepolizumab treatment to the time of each follow-up examination and also the last examination (*p* < 0.01; Fig. 5a, b). The relapse rate from the time of diagnosis to 1 year or more before mepolizumab administration, or within the year before the start of mepolizumab administration, did not differ between the two groups. Relapse rates every year after the start of mepolizumab treatment, and at the time of final examination, were significantly lower in super-responders than in responders (*p* < 0.01; Table 2 and Fig. 6). In super-responders, relapse rates were significantly lower than those at the start of mepolizumab treatment in each of the 3 years (*p* < 0.01) and at the time of the last visit (*p* < 0.01) (Fig. 6). Relapse rates after the start of administration of mepolizumab in responders did not change significantly compared with those at the start of mepolizumab (Fig. 6).

At our hospital, one of 23 patients who underwent conventional treatment had encephalopathy due to *Listeria monocytogenes.* One of 6 patients undergoing initial treatment had gastrointestinal involvement, and three of 43 patients (7.0%) treated with mepolizumab (average age 78.3 years) died of aspiration pneumonia or bacterial pneumonia.

## Discussion

IL-5 is a critical cytokine for the growth, maturation, and differentiation of eosinophils, making it an attractive target in EGPA treatment [50]. Mepolizumab, a humanized monoclonal antibody targeting IL-5, was developed in the late 1990s [51], and the results of its first clinical trial for asthma were published in 2000 [52]. MIRRA was the first randomized controlled trial of mepolizumab for treating refractory or relapsing EGPA [25]. Other published studies support the use of mepolizumab for the induction and maintenance of remission in refractory, relapsing, or glucocorticoid-dependent EGPA [25, 53–56]. However, it has not been conclusively determined whether mepolizumab treatment is effective against various vasculitis signs in EGPA or in ANCA-positive cases [57].

We administered mepolizumab to EGPA patients in whom conventional therapy had failed to induce remission. We showed that there were super-responders and responders with respect to the efficacy of mepolizumab; super-responders were characterized by high peripheral blood eosinophil counts at diagnosis and high serum IgG levels before mepolizumab administration [28]. We also showed that mepolizumab effectively treated vasculitis symptoms in many organs [28].

Here, we confirmed that all patients for whom conventional therapy could not induce remission received some clinical benefit after being subsequently treated with mepolizumab; among them were several patients with cardiac involvement (Table 1). The period required for the effects of clinical improvement with mepolizumab to become apparent varied, with a mean and standard deviation of 3.4 ± 3.0 months, and about 34.9% of patients experienced improvement on mepolizumab within 1 month. In 8/43 (18.6%) patients it took at least half a year for the improvement to become apparent (Fig. 2). We therefore consider that it is necessary to confirm any improvements after at least 1 year of administration, because a substantial period of time may be required for mepolizumab to take effect.

In one study, a group with a peripheral blood eosinophil count of  ≥ 150 cells/μL before administration of mepolizumab achieved remissions lasting 24 weeks, compared with a group with a peripheral blood eosinophil count of < 150 cells/μL [58]. In that study, and in our previous study [28], the peripheral blood eosinophil count decreased between the time of diagnosis and the time of initiation of mepolizumab, and it had decreased further in both of our groups by the time of last examination. We considered that the peripheral blood eosinophil count after the start of treatment was affected by the amounts of steroids and immunosuppressants administered up to that point. The treatment-naïve eosinophil count is a predictor of mepolizumab efficacy [28], and this is supported by the significantly higher blood eosinophil count in the super-responders at diagnosis (see Table 2). We confirmed the expectation that the mechanism underlying the clinical effects of mepolizumab leads to a decrease in the eosinophil count.

Our analysis also showed that the super-responders had a significantly higher peripheral blood eosinophil count at the time of diagnosis and a significantly higher serum IgG value before mepolizumab than the responders. We reported previously that EGPA patients with repeated relapses have activated CD86^+^ B cells and a decreased number of CD19^+^ B cells. As a result, serum IgG levels in these patients decrease and are not correlated with the concurrent PSL dose [59]. Our finding that serum IgG levels were maintained in the super-responders might reflect these patients’ ability to maintain their immune function; the effects of therapeutic drugs such as mepolizumab might thus be expressed better than in the responder group with low serum IgG.

Rates of manifestation of vasculitis signs, such as arthralgia or myalgia, were higher at diagnosis in the responders than in the super-responders (Table 2); we consider that these results reflect the small sample size of the analysis. In both groups, the BVAS at diagnosis was significantly lower both before and after mepolizumab administration than at diagnosis (Fig. 5). However, the BVAS values both before starting mepolizumab and at the time of the last visit were significantly lower in super-responders than in responders (Table 2). Most patients in the responder group had received immunosuppressants before administration of mepolizumab, and the PSL dose at their last visit on mepolizumab was significantly higher than that in the super-responder group.

There have been reports that mepolizumab is effective for vascular symptoms as manifested by asthma, arthralgia, and multiple polyneuropathy in EGPA patients [21, 25, 53–56, 60], but there have been no reports regarding its effect on long-term prognosis. In the super-responder group, the relapse rate was significantly lower in the third year and at the last visit (mean and standard deviation, 4.3 ± 1.0 years after the start of mepolizumab) than in the first year after the start of mepolizumab administration (Fig. 6). These results indicate that mepolizumab administration might reduce the relapse rate in the long term and thus improve the prognosis.

On the other hand, in the responder group, the BVAS decreased after the start of mepolizumab, but a reduction in the PSL daily dose was difficult to achieve in the long term, and the relapse rate after prolonged mepolizumab administration did not decrease. Thus, in responders considered to have severe and intractable disease, it is difficult to reduce the daily dose of steroids or other immunosuppressive agents; even if mepolizumab has some effect on vascular symptoms, it is unable to induce long-term remission.

We reported previously that the prognosis of EGPA could be improved by IVIG [15] via an increase in either the number of CD4^+^CD25^+^ T cells producing IL-10 [61] or the number of FOXP3^+^ regulatory T cells [62]. IVIG was administered to 22 of our 25 (88.0%) patients treated with mepolizumab (see Table 2), and it might induce remission by increasing the number of FOXP3 + regulatory T cells [61–63]. IL-33 and ILC2 are involved in the pathogenesis of asthma [64] and EGPA [9, 65]. We previously reported that, in EGPA, levels of ILC2 and IL-33 were high during onset and relapse and low during remission [9]. In addition, we reported that, in super-responders, the interval between IVIG treatments after mepolizumab initiation was significantly longer than that before mepolizumab [28].

We have also reported that the number of eosinophils in the colonic mucosa is significantly correlated with the number of Th17 cells in blood (CD4^+^ T cells producing IL-17) [48]. Increases in serum IL-17 and IL-22 levels are related to granuloma formation in sarcoidosis [66]. EGPA combines features of both hypereosinophilic disorders (Th2 activity) and ANCA-associated vasculitis (Th1 activity) [8]. In granulomatosis with polyangiitis, Th17 lymphocytes are a possible pathogenetic subset, and Treg cells are possible suppressors of the inflammatory process [67]. On the basis of these and our current results, we conclude that the synergistic effect of multiple drugs, including IVIG, mepolizumab, steroids, and other immunosuppressants, may have durably stabilized immune function and thus durably decreased the relapse rate in the super-responder group.

Biologics may trigger the production of antidrug antibodies against the drug, although one study did not detect any neutralizing antibodies [68]. There are cases of patients with asthma in whom the efficacy of mepolizumab has been attenuated over time [69]. However, long-term (> 10 years) administration of omalizumab has been reported in asthma [37] and there have been no reports of tolerance. Although omalizumab is the asthma biologic that has been on the market for the longest time, 'it is not clear when, or whether, its use should be discontinued in individual patients [70].

## Conclusion

Similarly, there are as yet no reports of the optimum administration duration or discontinuation timing of mepolizumab for EGPA. However, our results show that mepolizumab has long-term clinical efficacy and may improve prognosis in a subset of patients. A prospective study will be needed in future to confirm these findings.

## Data Availability

Not applicable.

## Abbreviations

EGPA: Eosinophilic granulomatosis with polyangiitis
BVAS: Birmingham vasculitis activity score
Il: Interleukin
ILC2: Innate lymphoid cell
IVIG: Intravenous immunoglobulin
MIRRA: Mepolizumab in relapsing or refractory EGPA
MPO-ANCA: Myeloperoxidase-antineutrophil cytoplasmic antibody
PR3: Protein 3
PSL: Prednisolone
Th: T-helper
